# P-1476. Safety of Homologous or Heterologous Priming and Booster Vaccinations with H5N8 or H5N6 MF59-Adjuvanted Cell Culture-derived Influenza Vaccine in Healthy Subjects ≥18 Years of Age

**DOI:** 10.1093/ofid/ofaf695.1662

**Published:** 2026-01-11

**Authors:** Adam Brosz, Janine Oberije, Eve Versage, Esther Van Twuijver, Matthew Hohenboken

**Affiliations:** Velocity Research, Grand Island, Nebraska; CSL Seqirus, Amsterdam, Noord-Holland, Netherlands; CSL Seqirus USA, Waltham, Massachusetts; CSL Seqirus, Amsterdam, Noord-Holland, Netherlands; CSL Seqirus, Amsterdam, Noord-Holland, Netherlands

## Abstract

**Background:**

Avian influenza is a global threat. This study assessed safety of a homologous and heterologous prime-boost regimen with MF59 adjuvanted, cell culture-derived H5N8 or H5N6 influenza vaccine (aH5N8c, aH5N6c) in adults aged ≥18 years.

**Figure:**
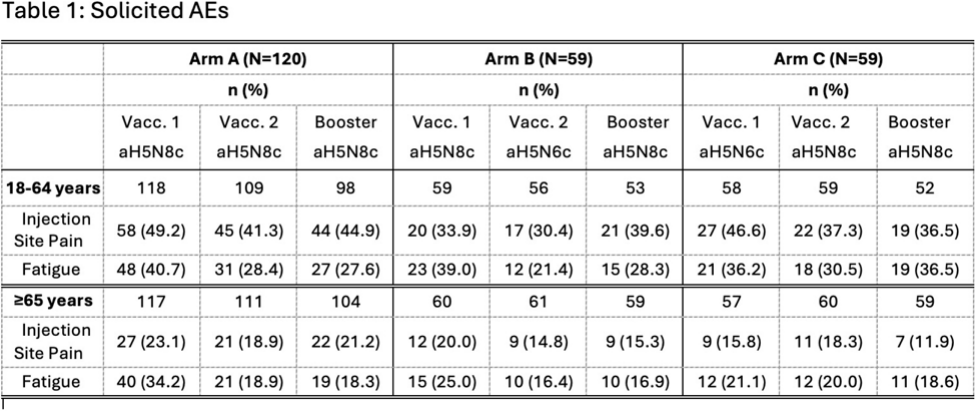

**Methods:**

In a Phase 2, randomized, observer-blind, multicenter study, adults 18-64 years (n=240) or ≥65 years (n=239) were randomized 2:1:1 as follows: Arm A: aH5N8c Days 1, 22; Arm B: aH5N8c Day 1, aH5N6c Day 22; Arm C: aH5N6c Day 1, aH5N8c Day 22. All subjects received aH5N8c booster on Day 202 (6 months post-2^nd^priming). Safety endpoints included solicited local and systemic adverse events (AEs), unsolicited AEs, serious AEs (SAEs), AEs of special interest (AESI), AEs leading to study withdrawal, and medically attended AEs (MAAEs). Data were analyzed by vaccination and age cohort.

**Results:**

Rates of solicited and unsolicited AEs were comparable among the 3 vaccine groups. Most solicited AEs (up to 7 days post-vaccination) were mild or moderate, occurred soon after vaccination, and resolved within 3 days. Rates of solicited AEs after the second priming and booster vaccinations were generally similar to or lower than rates after the first priming vaccination. Solicited AEs were reported by fewer subjects aged ≥65 than 18–64 years. The most frequent solicited local AE was injection site pain and the most frequent systemic AE was fatigue (Table 1). Rates of related unsolicited AEs (up to 3 weeks post-vaccination) were low in all treatment groups. MAAEs were reported by 36.7–45.8% of subjects over the course of 12 months, with no reports of related SAEs, AESIs, or deaths. One subject each from Arms A and B withdrew due to an AE.

**Conclusion:**

Multiple vaccinations with MF59 adjuvanted H5 vaccines (aH5N8c and aH5N6c) demonstrated an acceptable safety profile. Solicited AEs were common but mild or moderate and transient, with lower reactogenicity in older adults. Rates of solicited AEs did not increase after a second or third vaccine dose. The overall reactogenicity profile for aH5N8c/aH5N6c was consistent with observations from other MF59 adjuvanted pandemic influenza vaccine studies. No safety concerns were identified in this study.

Acknowledgement: This project received federal funds from The Center for the Biomedical Advanced Research and Development Authority (BARDA)

**Disclosures:**

Adam Brosz, MD, CSL Seqirus: Advisor/Consultant Janine Oberije, PhD, CSL Seqirus: Employee Eve Versage, MA, CSL Seqirus: Employee Esther Van Twuijver, PhD, CSL Seqirus: Employee Matthew Hohenboken, MD, PhD, CSL Seqirus: Employee

